# Parturition Synchrony Index: A Method for Assessing Individual Parturition Synchrony Within a Group or Population

**DOI:** 10.1002/ece3.72880

**Published:** 2026-01-02

**Authors:** Adam Dušek, Luděk Bartoš, Jitka Bartošová

**Affiliations:** ^1^ Department of Ethology, Institute of Animal Science Prague Czech Republic

**Keywords:** date of birth, inter‐individual variability, mammals, reproductive phenology, simulation, timing

## Abstract

The phenomenon of parturition synchrony at the population level has been studied for decades using various methods. To assess the tendency of individual mothers to synchronize their parturition timing with others, we developed a novel parturition synchrony index (PSI). The PSI quantifies an individual mother's degree of parturition synchrony within a group or population by detecting variation in the synchrony of parturitions, regardless of their actual timing within the season. A value of 1 indicates complete synchrony (i.e., a parturition occurring on the same day as others), whereas values approaching 0 reflect increasing asynchrony. To evaluate the robustness of the PSI, we conducted simulations examining how parturition‐date distribution (completely synchronous, lognormal, normal, bimodal, and uniform), parturition‐season duration (1, 10, 50, 100, 200, and 365 days), and group size (5, 10, 25, 50, 100, 500, 1000, and 5000 mothers) influence the PSI and its inter‐individual variability. A PSI value was calculated for each of the 140,490 individual mothers in the 168 simulated groups. For each group, we subsequently calculated both the mean PSI and the coefficient of variation (CV) of PSI values. All examined parameters influenced both metrics. Across all distributions (excluding complete synchrony), mean PSI values were higher in shorter than in longer parturition seasons. However, only in lognormal and normal distributions were mean PSI values markedly higher in larger than in smaller groups. Conversely, the CV was higher in longer seasons and in smaller groups across all distributions. Our results highlight the PSI's broad applicability to diverse socio‐ecological contexts. However, its reliability decreases under constrained conditions, such as very short seasons or extremely small groups, where wider confidence intervals were observed. The PSI thus provides a robust and flexible tool for quantifying and functionally analyzing individual parturition synchrony in mammals, and potentially in other viviparous vertebrates.

## Graphical abstract

We developed a novel parturition synchrony index (PSI) to quantify the degree of parturition synchrony for an individual mother within a group (or population). We evaluated the robustness of the PSI by simulating parturition synchrony at the individual level under a range of scenarios. The PSI provides a robust and flexible tool for quantifying and functionally analyzing individual parturition synchrony in mammals and potentially in other viviparous vertebrates.

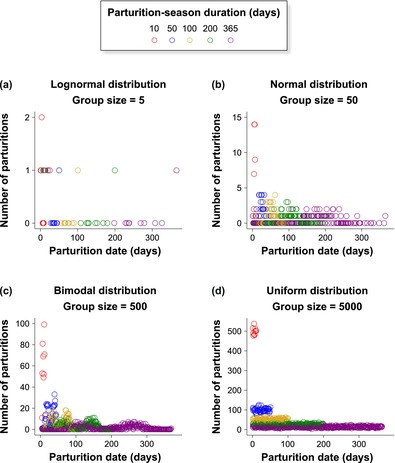

## 1 Introduction

The majority of mammals inhabit seasonal environments and regulate their reproductive cycles, including the timing of parturition, to ensure that offspring are reared under the most favorable ecological conditions throughout the year (Bronson and Heideman 1994). Timing of parturition largely determines the temporal clustering of births (reviewed by Ims 1990), which in turn can affect growth (e.g., in the roe deer *Capreolus capreolus* ; Plard et al. 2015), survival (e.g., in the banded mongoose *Mungos mungo* ; Hodge et al. 2011), and recruitment of individual offspring (e.g., in the wood lemming *Myopus schisticolor* ; Ims et al. 1988).

The phenomenon of parturition synchrony, defined as the temporal alignment of births within a group or population, is regarded as an evolutionary adaptation to various selective pressures. These pressures include: (1) optimizing birth timing to maximize offspring growth and survival in response to seasonal changes in the environment, as observed in ungulates (Rutberg 1987; Zerbe et al. 2012), seals (Atkinson 1997), bats (Eghbali and Sharifi 2023), and carnivores (Mattisson et al. 2022); (2) minimizing predation risk by producing large numbers of precocial newborns within a short period of time, leading to predator saturation or confusion, as evidenced in follower ungulates (Rutberg 1987; Sinclair et al. 2000) and squirrel monkeys (Boinski 1987); or (3) facilitating the shared thermoregulation of developing young, as observed in social bats (Heideman and Utzurrum 2003). Similarly, communal breeding has shaped the evolution of parturition synchrony in some mammals, such as the house mouse ( *Mus musculus* ), where synchronized births prevent infanticidal behavior among nestmates (Schmidt et al. 2015). This breeding system has also had a profound impact on the reproductive behavior of the banded mongoose, a species that exhibits extreme parturition synchrony, with up to 64% of births occurring in a single night (Hodge et al. 2011). This remarkable tactic enables the reduction of the risk of infanticide and the minimization of sibling competition within the group. Even in mixed‐species groups, such as the hyraxes *Procavia capensis* and *Heterohyrax brucei* , which occupy the same territory, females may synchronize parturitions to optimize parental care (Barry and Mundy 2002).

However, in mammals with socially structured populations, individual mothers often display substantial variation in the degree of parturition synchrony. This variation is shaped by multiple factors, including maternal age (Boltnev and York 2001; Plard et al. 2014; Rotella et al. 2016; Stopher et al. 2008), reproductive status during the previous breeding season (Adams and Dale 1998; Guinness et al. 1978; Rotella et al. 2016), body mass (Birgersson and Ekvall 1997; Boltnev and York 2001; Loe et al. 2005; Plard et al. 2014), condition (Berger 1992; Robbins et al. 2012; Rotella et al. 2016), and social rank (Cant et al. 2014; Dezeure et al. 2022; Holand et al. 2004; Stopher et al. 2008). Maternal traits such as age, condition, and social rank can further enhance fitness by facilitating the synchronization of parturition within the group (see, e.g., Asher 2007; Berger 1992; Cant et al. 2014; Scott et al. 2008). Specifically, synchronized parturition enhances offspring survival and fitness in numerous social species by enabling communal parental care (Russell and Lummaa 2009) and facilitating coordinated antipredator behaviors (Gaillard et al. 2000; Rutberg 1987). Consequently, individual variation in maternal quality can affect both parturition synchrony and reproductive success at the population level (i.e., the number of surviving offspring produced in a single reproductive cycle).

Although inter‐individual variation in parturition synchrony is a well‐documented phenomenon in mammals, prior research has focused on assessing this variation at the group or population level. Current methods for assessing parturition synchrony include calculating the proportion of offspring born within defined time intervals (Adams and Dale 1998; Ogutu et al. 2015; Sinclair et al. 2000), measuring the coefficient of variation (CV) of birth dates within a geographic region (Mattisson et al. 2022; Michel et al. 2020), applying Poisson regression models (Ogutu et al. 2010), and utilizing circular statistics, such as the mean vector length (Paré et al. 1996; Thel et al. 2022). Despite the notable advancements in this field, no standardized method exists for quantifying parturition synchrony at the individual level within groups or populations. This methodological gap is largely due to the logistical challenges of simultaneously monitoring parturition timing at both the individual and population levels (but see DeMars et al. 2013; Marchand et al. 2021; Turnley et al. 2024, for related approaches). In this article, we propose a new method for assessing the degree of parturition synchrony for an individual mother within a group or population. Our method builds on earlier studies (Langefors et al. 1998; Marsden and Evans 2004; Stutchbury et al. 1998) that examined inter‐individual variation in breeding synchrony among females during their fertile periods.

To evaluate the robustness of the proposed method and its potential applicability across diverse socio‐ecological contexts, we simulated parturition synchrony under a range of scenarios defined by three key socio‐ecological parameters: parturition‐date distribution, parturition‐season duration, and group size.

## 2 Materials and Methods

### 2.1 Parturition Synchrony Index

At the individual level, we define parturition synchrony as the tendency of a mother to give birth at the same time (*t*) as other mothers within the same group (or population). For each mother *m*, parturition synchrony is calculated as a function of all parturition dates within the group, specifically as the reciprocal of the mean difference between the parturition date of mother *m* and the parturition dates of the other mothers. The parturition synchrony index (PSI) for mother *m* can thus be expressed as follows:

(1) $${\mathrm{PSI}}_{m}=\frac{1}{\frac{\sum_{\begin{array}{c} i=1\\ i\neq m \end{array}}^{N}\left[\left|t_{i}-t_{m}\right|+1\right]}{N-1}},$$

where *N* is the total number of parturient mothers in the group; *t*_*m*_ is the relative parturition date of mother *m* (i.e., the number of days elapsed since the first mother in the group gave birth within a given parturition season; e.g., if the first parturition occurred on May 30th, then *t*_*m*_ = 1); and *t*_*i*_ is the relative parturition date of any other mother within the group (*i* = 1, 2, 3, …; *N* − 1; *i* ≠ *m*). The absolute value of the difference between *t*_*m*_ and *t*_*i*_ provides a measure of the relative parturition date of mother *m* during the birthing period. This measure emphasizes inter‐individual variation in the degree of parturition synchrony, regardless of whether mother *m* gave birth earlier or later than any other mother *i* within the group (i.e., the difference between *t*_*m*_ and *t*_*i*_ remains the same, whether *t*_*m*_ < *t*_*i*_ or *t*_*m*_ > *t*_*i*_). Consequently, the PSI value of a first‐birthing mother may be equal to that of a last‐birthing mother within the same season.

The PSI represents the degree to which the parturition date of mother *m* is synchronized with the parturition dates of all other mothers within the group. A higher value indicates a higher degree of parturition synchrony for the given mother. Under complete synchrony, when all mothers within the group give birth on the same day, the value of |*t*_*i*_ − *t*_*m*_| (see Equation 1) is 0, which results in both the denominator and the PSI being equal to 1. Conversely, under asynchronous parturition, as the denominator increases, the PSI value asymptotically approaches 0. However, the PSI is always > 0 because it reflects differences between the relative parturition dates of at least two mothers. Therefore, the PSI provides a measure of inter‐individual variation in parturition synchrony among mothers.

We quantify the degree of group parturition synchrony by calculating the mean PSI, which is defined as:

(2) $$\bar{\mathrm{PSI}}=\frac{1}{N}\;\sum_{m=1}^{N}\;\left[\frac{1}{\frac{\sum_{\begin{array}{c} i=1\\ i\neq m \end{array}}^{N}\left[\left|t_{i}-t_{m}\right|+1\right]}{N-1}}\right].$$

The value of $\bar{\mathrm{PSI}}$ represents the mean degree of parturition synchrony across all mothers within the group. In this variant of the index, a higher value indicates a higher degree of parturition synchrony within the group.

### 2.2 Data Simulation and Analysis

We conducted all data simulations and analyses using the SAS System V 9.4 (SAS Institute, Cary, NC, USA).

**FIGURE 1:**
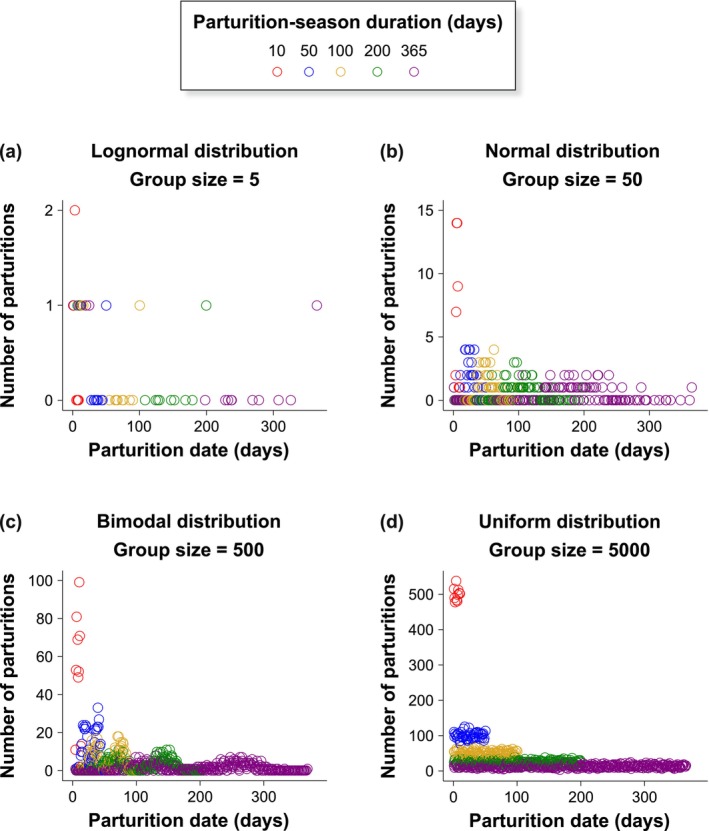
Number of parturitions by simulated parturition date across all examined parturition‐season durations (10, 50, 100, 200, and 365 days) in four example scenarios combining parturition‐date distributions and group sizes: (a) lognormal distribution, 5 mothers (*N* = 25); (b) normal distribution, 50 mothers (*N* = 250); (c) bimodal distribution, 500 mothers (*N* = 2500); and (d) uniform distribution, 5000 mothers (*N* = 25,000).

The robustness of the PSI was evaluated by simulating parturition dates across groups defined by unique combinations of three parameters: (1) Parturition‐date distribution (five types: Completely synchronous—with all parturitions occurring on the same day; Lognormal; Normal; Bimodal—generated from two normal distributions with peaks always at 30% and 70% of the individual parturition‐season duration, for example, in a 200‐day season, the peaks occur on days 60 and 140; and Uniform); (2) Parturition‐season duration (six durations: 1, 10, 50, 100, 200, and 365 days); and (3) Group size (eight sizes: 5, 10, 25, 50, 100, 500, 1000, and 5000 mothers). For each combination of these parameters, we simulated 40 independent groups, except for the completely synchronous scenario, where the season duration was fixed at 1 day, resulting in only eight groups (one for each group size). In total, we generated 140,490 parturition dates, each corresponding to an individual mother, across 168 simulated groups. This includes 6690 dates in the completely synchronous distribution and 33,450 dates in each of the other distributions (see Figure 1 for illustrative examples).

To ensure internal consistency and control over the key data set properties, we first generated a preliminary data set of 100,000 randomly simulated parturition dates for each relevant combination of Parturition‐date distribution and Parturition‐season duration using the RAND function (STREAMINIT = 777). We then applied the PROC SURVEYSELECT procedure (seed = 777) to randomly sample the number of dates required to match the target group size. Each group spanned the full duration of the parturition season, including both the first and last day (e.g., days 1 and 100 for a 100‐day season). Finally, we calculated PSI values for all individual mothers within the simulated groups (see Table S1.1, Simulated data set sheet, Appendix S1).

Descriptive statistics for the PSI, including the minimum, maximum, mean, median, standard deviation, and CV, were calculated using the PROC UNIVARIATE procedure (see Table S1.1, Descriptive statistics sheet, Appendix S1). Variation in the PSI across 40 simulated groups within each examined distribution was subsequently visualized using box plots (see Figure S2.1, Appendix S2).

To assess the effects of the tested parameters on the PSI, we used the $\bar{\mathrm{PSI}}$ and the CV of the PSI as dependent variables, both calculated for each simulated group, excluding the completely synchronous distribution (*N* = 160 groups). First, we tested the assumption that the Parturition‐date distribution affects the $\bar{\mathrm{PSI}}$ and the CV of the PSI. Second, after confirming this effect, we investigated how the Parturition‐season duration and Group size influenced these metrics, with both factors nested within the Parturition‐date distribution. Analyses were conducted using a general linear model (GLM) with maximum likelihood estimation implemented in the PROC GLIMMIX procedure. Each fixed effect was estimated while controlling for the others, thereby holding all remaining fixed effects constant. Model fit was assessed through visual inspection of residual normality, randomness of error terms, and homoscedasticity. The significance of fixed effects was determined using *F*‐tests. Within‐class differences were represented by least‐squares (LS) means with 95% confidence intervals (CIs). The LS means were numerically identical to the arithmetic means across all factor levels. All *p‐*values were two‐tailed, with a significance level (*α*) of 0.05.

## 3 Results

The PSI values ranged from 0.003 to 1, with both the $\bar{\mathrm{PSI}}$ and the CV of the PSI showing substantial variation among the examined distributions (see Table S1.1, Descriptive statistics sheet, Appendix S1). As expected, no inter‐individual variability was observed in the completely synchronous distribution, in which all individuals had identical, maximum PSI values (mean = 1, CV = 0). More generally, variation in the PSI across distributions further depended on parturition‐season duration and group size (see Figure S2.1, Appendix S2).

**FIGURE 2:**
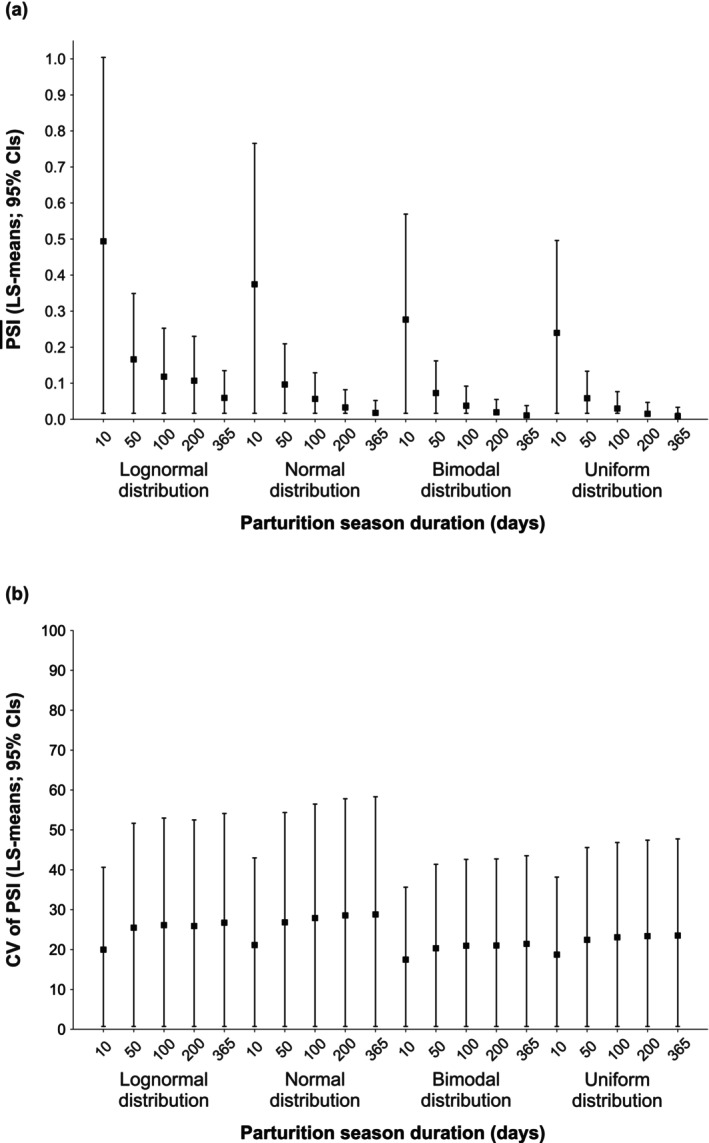
Effect of parturition‐season duration on (a) the $\bar{\mathrm{PSI}}$ (LS‐means; 95% CIs) and (b) the CV of the PSI (LS‐means; 95% CIs) across simulated parturition‐date distributions (lognormal, normal, bimodal, and uniform). For each distribution, an equal number of PSI values (*N* = 6690) was used at each examined parturition‐season duration (10, 50, 100, 200, and 365 days).

**FIGURE 3:**
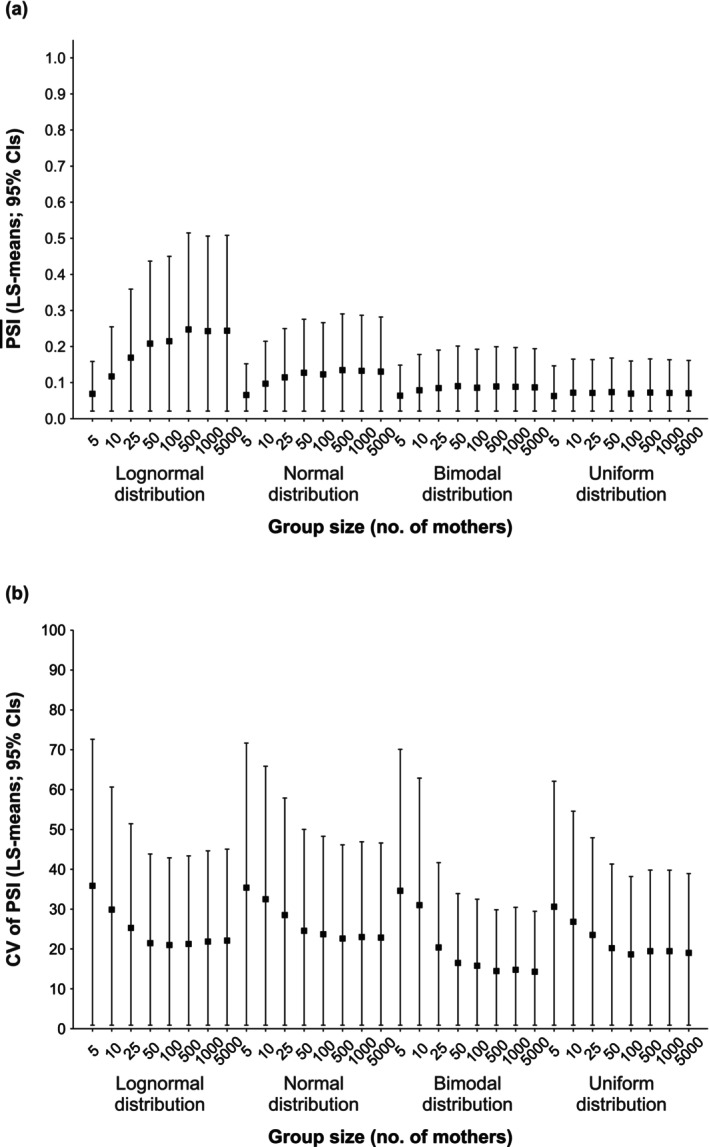
Effect of group size on (a) the $\bar{\mathrm{PSI}}$ (LS‐means; 95% CIs) and (b) the CV of the PSI (LS‐means; 95% CIs) across simulated parturition‐date distributions (lognormal, normal, bimodal, and uniform). For each distribution, a predefined number of PSI values was used at each group size (5 mothers: *N* = 25; 10 mothers: *N* = 50; 25 mothers: *N* = 125; 50 mothers: *N* = 250; 100 mothers: *N* = 500; 500 mothers: *N* = 2500; 1000 mothers: *N* = 5000; and 5000 mothers: *N* = 25,000).

The $\bar{\mathrm{PSI}}$ differed among the distributions of parturition dates in the simulated groups (GLM: *F*_(3,156)_ = 6.75, *p* < 0.001). Among the examined distributions, the lognormal distribution showed the highest $\bar{\mathrm{PSI}}$ (LS‐mean = 0.19; 95% CI: 0.15–0.23), followed by the normal (LS‐mean = 0.12; 95% CI: 0.08–0.16), the bimodal (LS‐mean = 0.08; 95% CI: 0.04–0.12), and the uniform (LS‐mean = 0.07; 95% CI: 0.03–0.11) distributions. Furthermore, the $\bar{\mathrm{PSI}}$ differed according to parturition‐season duration (GLM: *F*_(16,112)_ = 268.59, *p* < 0.0001), tending to be lower in longer than in shorter durations (Figure 2a). This trend was observed across all distributions, most notably under the lognormal distribution, where narrow CIs indicated a robust effect. Group size also influenced the $\bar{\mathrm{PSI}}$ (GLM: *F*_(28,112)_ = 11.30, *p* < 0.0001; Figure 3a), although the magnitude of this effect varied among distributions. Notably, the lognormal distribution showed substantial variation in the $\bar{\mathrm{PSI}}$ across group sizes, with overlapping CIs in smaller groups (5–25 mothers).

The distribution of parturition dates also affected the CV of the PSI (GLM: *F*_(3,156)_ = 8.74, *p* < 0.0001). Among the examined distributions, the normal distribution had the highest CV (LS‐mean = 26.64; 95% CI: 24.75–28.53), followed by the lognormal (LS‐mean = 24.84; 95% CI: 22.95–26.72) and the uniform (LS‐mean = 22.22; 95% CI: 20.33–24.11) distributions, whereas the bimodal distribution showed the lowest CV (LS‐mean = 20.23; 95% CI: 18.35–22.12). Within each distribution, the CV varied according to both parturition‐season duration (GLM: *F*_(16,112)_ = 48.40, *p* < 0.0001) and group size (GLM: *F*_(28,112)_ = 172.20, *p* < 0.0001). The CV was lowest for the 10‐day parturition‐season duration and tended to increase with longer season durations, which were accompanied by wider CIs (Figure 2b). It was also higher in smaller than in larger group sizes, with smaller groups showing correspondingly wider CIs (Figure 3b).

## 4 Discussion

The PSI facilitates the quantification of parturition synchrony at the individual level. Using simulated data, we demonstrated that parturition‐date distribution, season duration, and group size influenced two key components of parturition timing: individual synchrony and inter‐individual variability. While the effect of parturition‐season duration on the PSI was relatively straightforward, the influences of parturition‐date distribution and group size were more complex and context‐dependent. Nevertheless, all three parameters shaped the PSI in biologically meaningful ways and warrant consideration as true biological factors rather than mere artifacts. To clarify these dynamics, we interpret the observed patterns within the evolutionary ecology framework, drawing on empirical studies of model mammalian taxa.

### 4.1 Influence of Parturition‐Season Duration on PSI

As expected, the $\bar{\mathrm{PSI}}$ decreased from shorter to longer parturition‐season durations across all tested distributions, with the steepest decline between 10‐ and 50‐day parturition seasons (Figure 2a). This pattern aligns with theoretical predictions that longer breeding seasons relax temporal constraints on births, thereby reducing reproductive synchrony (Ims 1990), and mirrors interspecific trends in wild ruminants, where narrower seasonal windows yield tighter clustering of parturition (Rutberg 1987). The concurrent narrowing of the PSI CIs, particularly between 10 and 100 days, reinforces the robustness of this relationship. Although wide CIs for the shortest (10‐day) seasons indicate greater uncertainty in the $\bar{\mathrm{PSI}}$ estimates, the overall downward trend persisted across all distributions. These findings reveal a systematic, albeit nonlinear, association between season duration and the PSI, highlighting the index's sensitivity and its potential utility in systems where seasonal environmental constraints shape reproductive phenology (as reviewed by Varpe 2017).

The sharp decline in the $\bar{\mathrm{PSI}}$ values between days 10 and 50 likely reflects the increasing inter‐individual variation in parturition timing (Figure 2b). Despite the wider CIs of the CV of the PSI at longer durations, the directional trend remained robust. This suggests that the PSI can detect inter‐individual differences in parturition synchrony even when its timing is more dispersed, multimodal, or asynchronous. These simulation‐based patterns closely align with empirical evidence of intraspecific phenotypic plasticity in reproductive phenology (see Nussey et al. 2007, for a review).

Collectively, these findings highlight the utility of the PSI in detecting individual‐level parturition synchrony across diverse seasonal contexts. Greater variability in individual PSI values among mothers during longer seasons reflects increased divergence in parturition timing as the temporal window for giving birth expands. This suggests that maternal identity has a more substantial influence on synchrony patterns under flexible reproductive phenologies, as observed, for example, in chacma baboons ( *Papio ursinus* ; Dezeure et al. 2021). Accordingly, the PSI provides a robust and versatile metric for quantifying individual contributions to parturition synchrony across groups, populations, and species with varying parturition‐season durations.

### 4.2 Maternal Group Size Effects on PSI

In line with our predictions, group size influenced PSI values. Specifically, smaller groups of five mothers showed lower $\bar{\mathrm{PSI}}$ values than larger groups of ≥ 50 for both lognormal and normal distributions (Figure 3a). Nonetheless, the consistently narrow CIs of the $\bar{\mathrm{PSI}}$ in small groups across all distributions indicate that the PSI reliably estimates individual‐level parturition synchrony, even when group size is very small. This pattern reflects greater inter‐individual variability in smaller groups (Figure 3b). Such variability likely arises from more substantial individual‐level effects under demographic constraints (Lande et al. 2003, 10, 15–16; Vindenes et al. 2008), where individual PSI values exert a proportionally greater influence on the $\bar{\mathrm{PSI}}$ in smaller groups. However, the consistently wide CIs of the CV of the PSI in smaller groups suggest that researchers should interpret PSI patterns cautiously in this size category, where stochastic variation among individuals may exert a disproportionate influence.

These results align with empirical trends observed across wild mammalian populations. For instance, in communally breeding degus ( *Octodon degus* ), smaller groups show greater variability in parturition timing (Matchinske et al. 2024), and in sympatric hyraxes, larger groups display higher parturition synchrony (Barry and Mundy 2002). Furthermore, in wildebeest ( *Connochaetes taurinus* ), the positive association between group size and parturition synchrony depends on environmental context: large, nomadic groups inhabiting areas with seasonally or irregularly available water and forage exhibit more synchronized parturitions than smaller, resident groups of approximately 10 females occupying habitats with perennially available resources (Estes 1966, 1976; Ndibalema 2009).

Together, these observations support group size as a key driver of parturition synchrony across social systems. Furthermore, they highlight the PSI's value as a sensitive, consistent metric in detecting individual‐level contributions to parturition timing in diverse social structures.

### 4.3 PSI Variation Across Parturition‐Date Distributions

Our results show that the PSI is sensitive to variation in the shape of parturition‐date distributions. These findings are consistent with both theoretical models (Burtschell et al. 2023) and empirical observations (reviewed by Zerbe et al. 2012), and have important implications for its interpretation and application. Specifically, differences in both the $\bar{\mathrm{PSI}}$ and the CV of the PSI across all tested distributions suggest that characteristics such as skewness, modality, and spread may influence temporal patterns of individual parturition synchrony. However, despite this sensitivity, the relatively narrow CIs indicate that the PSI captures individual‐level variation in parturition synchrony while still providing stable group‐level estimates. This robustness allows it to reliably distinguish temporal structures of parturition—a feature often lacking in earlier, inconsistent or study‐specific metrics (Thel et al. 2022).

### 4.4 Socio‐Ecological Implications of PSI

Consistent with recent empirical research (Matchinske et al. 2024; Philson et al. 2024), our findings indicate a causal influence of socio‐ecological factors on intragroup parturition synchrony. Specifically, the observed variation in the PSI across parturition‐season durations, group sizes, and parturition‐date distributions indicates that the index is sensitive to these factors, supporting its potential to capture individual‐level synchrony patterns within groups across taxa exhibiting varying degrees of seasonality and sociality. Furthermore, markedly higher PSI values in shorter parturition seasons (Figure 2a) suggest that the PSI may be particularly effective for detecting parturition synchrony in seasonal, socially structured systems, where mothers vary substantially in both the quality and timing of parturition (e.g., Adams and Dale 1998; Dezeure et al. 2022; Hodge et al. 2011; Robbins et al. 2012).

The applicability of the PSI extends to two contrasting models of parturition synchrony. First, it is relevant for taxa in which females of similar quality give birth in synchrony during the parturition season, as documented in ruminants (Holand et al. 2004; Plard et al. 2014; Stopher et al. 2008), carnivores (Cram et al. 2019; Ordiz et al. 2008; Robbins et al. 2012), and pinnipeds (Boltnev and York 2001; Rotella et al. 2016). Second, the PSI may be informative in species where females of varying quality form smaller groups and give birth within compressed timeframes. In such systems, parturition synchrony may be driven by the presence or reproductive tactics of high‐quality group members, as observed in banded mongooses (Cant et al. 2014) and bison ( *Bison bison* ; Berger 1992). The PSI can thus be used to test whether individual parturition synchrony is influenced by maternal quality, offering insights into how individual traits shape parturition timing and enabling assessment of whether such synchrony is adaptively maintained within groups, as suggested for the red deer ( *Cervus elaphus* ; Asher 2007; Scott et al. 2008).

Moreover, in both systems, the PSI may serve as a valuable tool for testing how maternal synchrony influences individual behavioral traits. At the group level, it enables the quantification of how inter‐individual differences in parturition synchrony contribute to variation in seasonality (Dezeure et al. 2022), sociability (Matchinske et al. 2024; Philson et al. 2024), the degree of communal parental care (Russell and Lummaa 2009), and the effectiveness of anti‐predator tactics (Gaillard et al. 2000; Rutberg 1987). Ultimately, our approach may help elucidate the mechanisms driving variation in parturition synchrony across multiple hierarchical levels—from subgroups within groups to populations within species and related taxa.

### 4.5 Applications and Utility of PSI

The PSI is a flexible metric with broad relevance for functional and comparative analyses of individual parturition synchrony across mammalian taxa. Beyond quantifying inter‐individual variation in parturition timing, the PSI offers a tool to investigate how maternal traits—such as age, body mass, condition, social rank, or reproductive experience—influence a mother's degree of parturition synchrony with her group mates. This approach enables researchers to explore the potential fitness consequences of parturition synchrony, including inter‐individual differences in reproductive success.

Comparative application of the PSI requires researchers to take into account socio‐ecological variation across groups or populations, emphasizing inter‐individual variation in maternal parturition synchrony within each group. Factors such as parturition seasonality, group size, and the temporal structure of parturitions may influence PSI values independently of other socio‐ecological variation. Consequently, incorporating these influences into appropriate statistical models is essential for a robust assessment of individual‐level parturition synchrony.

The PSI offers a framework for investigating the mechanisms underpinning parturition synchronization, particularly the interplay between physiological and social drivers. For instance, it can be used to assess the extent to which parturition synchrony is influenced by social facilitation among mothers (as suggested by Rutberg 1987). Beyond this specific application, researchers can use the PSI to explore how geographic variation among populations shapes patterns of parturition synchrony. More broadly, the PSI may provide valuable insights into how parturition synchrony influences reproductive timing by promoting phenotypic plasticity at the individual level (Dezeure et al. 2022; Mattisson et al. 2022; Nussey et al. 2005) and driving evolutionary change at the population level (Bonnet et al. 2019).

From a practical perspective, this novel approach has potential applications in mammalian conservation, management, and breeding. For example, in seasonal breeders, the PSI could help determine the impact of environmental variability, resource availability, and predation risk on individual parturition synchrony and its subsequent effects on maternal care, offspring survival, and population dynamics. Alternatively, the PSI may serve to assess the influence of breeding conditions on variation in parturition synchrony and species' adaptation to environmental changes or photoperiodic cues in captivity. It may also help evaluate the success of practices such as cross‐fostering.

## 5 Conclusion

In summary, the PSI is a quantitative tool for assessing parturition synchrony at the individual level. A high PSI value indicates a greater degree of synchrony for a given mother within a group (or population), whereas a high $\bar{\mathrm{PSI}}$ value reflects greater synchrony across the group as a whole. The PSI is a versatile metric, applicable across various combinations of parturition‐date distributions, season durations, and group sizes. It reaches its highest values under lognormal distributions. Moreover, in both lognormal and normal distributions, PSI values tend to be higher in larger groups than in smaller ones. PSI values are also consistently higher during shorter parturition seasons, regardless of distribution type. Variability in the PSI increases with longer parturition seasons and smaller maternal groups. However, the index may be less reliable for detecting inter‐individual differences in synchrony under particularly constrained socio‐ecological conditions, such as very short seasons or extremely small groups. Despite these limitations, the PSI provides a novel and broadly applicable framework for quantifying and functionally analyzing parturition synchrony at the individual level in mammals, and potentially in other viviparous vertebrates.

## Author Contributions

**Adam Dušek:** conceptualization (lead), data curation (lead), formal analysis (equal), investigation (equal), methodology (equal), project administration (equal), resources (equal), software (equal), validation (equal), visualization (equal), writing – original draft (lead), writing – review and editing (lead). **Luděk Bartoš:** conceptualization (supporting), formal analysis (equal), investigation (equal), methodology (lead), project administration (lead), resources (equal), software (equal), supervision (equal), validation (equal), visualization (equal), writing – review and editing (equal). **Jitka Bartošová:** conceptualization (supporting), formal analysis (lead), funding acquisition (lead), investigation (equal), methodology (equal), project administration (equal), resources (equal), software (equal), supervision (equal), validation (equal), writing – review and editing (equal).

## Funding

This work was supported by the Ministry of Agriculture of the Czech Republic, institutional support MZE‐RO0723.

## Conflicts of Interest

The authors declare no conflicts of interest.

## Supporting information


**Appendix S1:** Table S1.1 showing the sheets “data description,” “simulated data set,” and “descriptive statistics.”

**Appendix S2:** Figure S2.1 showing variation in PSI values across simulated groups covering all combinations of examined parameters.

**Appendix S3:** Table S3.1 showing definitions of variables used and SAS and R code for calculating individual PSI values using an example data set.

**Appendix S4:** Table S4.1 showing the sheets “data description” and “PSI calculation tool” for the automated computation of PSI values for a sample group of mothers within a single parturition season.

## Data Availability

The data generated and analyzed in this study, along with supporting information, statistical code, and a PSI calculation tool, are provided in Appendices S1–, S4. Appendix S1 includes Table S1.1 with the simulated data set and descriptive statistics of the PSI calculated for each simulated group. Appendix S2 presents Figure S2.1, which shows box plots illustrating variation in PSI values across 40 simulated groups covering all combinations of season duration and group size under four different parturition‐date distributions. Appendix S3 contains the SAS and R code used to calculate PSI values for a sample group within a single parturition season. Finally, Appendix S4 provides Table  S4.1, which introduces a PSI calculation tool that automatically computes PSI values for a sample group of mothers within a single parturition season.

## References

[ece372880-bib-0001] Adams, L. G. , and B. W. Dale . 1998. “Timing and Synchrony of Parturition in Alaskan Caribou.” Journal of Mammalogy 79, no. 1: 287–294. 10.2307/1382865.

[ece372880-bib-0002] Asher, G. W. 2007. “Gestation Length in Red Deer: Genetically Determined or Environmentally Controlled?” Society of Reproduction and Fertility Supplement 64: 255–260. 10.5661/rdr-vi-255. 17491152

[ece372880-bib-0003] Atkinson, S. 1997. “Reproductive Biology of Seals.” Reviews of Reproduction 2: 175–194. 10.1530/revreprod/2.3.175. 9414481

[ece372880-bib-0004] Barry, R. E. , and P. J. Mundy . 2002. “Seasonal Variation in the Degree of Heterospecific Association of Two Syntopic Hyraxes (*Heterohyrax brucei* and *Procavia capensis*) Exhibiting Synchronous Parturition.” Behavioral Ecology and Sociobiology 52, no. 3: 177–181. 10.1007/s00265-002-0509-8.

[ece372880-bib-0005] Berger, J. 1992. “Facilitation of Reproductive Synchrony by Gestation Adjustment in Gregarious Mammals: A New Hypothesis.” Ecology 73, no. 1: 323–329. 10.2307/1938743.

[ece372880-bib-0006] Birgersson, B. , and K. Ekvall . 1997. “Early Growth in Male and Female Fallow Deer Fawns.” Behavioral Ecology 8, no. 5: 493–499. 10.1093/beheco/8.5.493.

[ece372880-bib-0007] Boinski, S. 1987. “Birth Synchrony in Squirrel Monkeys (*Saimiri oerstedi*): A Strategy to Reduce Neonatal Predation.” Behavioral Ecology and Sociobiology 21, no. 6: 393–400. 10.1007/BF00299934.

[ece372880-bib-0008] Boltnev, A. I. , and A. E. York . 2001. “Maternal Investment in Northern Fur Seals ( *Callorhinus ursinus* ): Interrelationships Among Mothers' Age, Size, Parturition Date, Offspring Size and Sex Ratios.” Journal of Zoology 254, no. 2: 219–228. 10.1017/S0952836901000735.

[ece372880-bib-0009] Bonnet, T. , M. B. Morrissey , A. Morris , et al. 2019. “The Role of Selection and Evolution in Changing Parturition Date in a Red Deer Population.” PLoS Biology 17, no. 11: e3000493. 10.1371/journal.pbio.3000493. 31689300 PMC6830748

[ece372880-bib-0010] Bronson, F. H. , and P. D. Heideman . 1994. “Seasonal Regulation of Reproduction in Mammals.” In Physiology of Reproduction, edited by E. Knobil and J. D. Neill , 2nd ed., 541–583. Raven Press.

[ece372880-bib-0011] Burtschell, L. , J. Dezeure , E. Huchard , and B. Godelle . 2023. “Evolutionary Determinants of Reproductive Seasonality: A Theoretical Approach.” Peer Community Journal 3: e56. 10.24072/pcjournal.286.

[ece372880-bib-0012] Cant, M. A. , H. J. Nichols , R. A. Johnstone , and S. J. Hodge . 2014. “Policing of Reproduction by Hidden Threats in a Cooperative Mammal.” Proceedings of the National Academy of Sciences 111, no. 1: 326–330. 10.1073/pnas.1312626111. PMC3890811 24367092

[ece372880-bib-0013] Cram, D. L. , A. Jungwirth , H. Spence‐Jones , and T. Clutton‐Brock . 2019. “Reproductive Conflict Resolution in Cooperative Breeders.” Behavioral Ecology 30, no. 6: 1743–1750. 10.1093/beheco/arz143.

[ece372880-bib-0014] DeMars, C. A. , M. Auger‐Méthé , U. E. Schlägel , and S. Boutin . 2013. “Inferring Parturition and Neonate Survival From Movement Patterns of Female Ungulates: A Case Study Using Woodland Caribou.” Ecology and Evolution 3, no. 12: 4149–4160. 10.1002/ece3.785. 24324866 PMC3853560

[ece372880-bib-0015] Dezeure, J. , A. Baniel , A. Carter , G. Cowlishaw , B. Godelle , and E. Huchard . 2021. “Birth Timing Generates Reproductive Trade‐Offs in a Non‐Seasonal Breeding Primate.” Proceedings of the Royal Society B 288, no. 1950: 20210286. 10.1098/rspb.2021.0286. 33975480 PMC8113908

[ece372880-bib-0016] Dezeure, J. , M. J. E. Charpentier , and E. Huchard . 2022. “Fitness Effects of Seasonal Birth Timing in a Long‐Lived Social Primate Living in the Equatorial Forest.” Animal Behaviour 185: 113–126. 10.1016/j.anbehav.2022.01.002.

[ece372880-bib-0017] Eghbali, H. , and M. Sharifi . 2023. “Impacts of Inter‐Annual Climate Variability on Reproductive Phenology and Postnatal Development of Morphological Features of Three Sympatric Bat Species.” Scientific Reports 13, no. 1: 8716. 10.1038/s41598-023-35781-6. 37248331 PMC10227034

[ece372880-bib-0018] Estes, R. D. 1966. “Behaviour and Life History of Wildebeest (*Connochaetes taurinus* Burchell).” Nature 212, no. 5066: 999–1000. 10.1038/212999a0.

[ece372880-bib-0019] Estes, R. D. 1976. “The Significance of Breeding Synchrony in the Wildebeest.” African Journal of Ecology 14, no. 2: 135–152. 10.1111/j.1365-2028.1976.tb00158.x.

[ece372880-bib-0020] Gaillard, J. M. , M. Festa‐Bianchet , N. G. Yoccoz , A. Loison , and C. Toïgo . 2000. “Temporal Variation in Fitness Components and Population Dynamics of Large Herbivores.” Annual Review of Ecology and Systematics 31, no. 1: 367–393. 10.1146/annurev.ecolsys.31.1.367.

[ece372880-bib-0021] Guinness, F. E. , R. M. Gibson , and T. H. Clutton‐Brock . 1978. “Calving Times of Red Deer ( *Cervus elaphus* ) on Rhum.” Journal of Zoology 185, no. 1: 105–114. 10.1111/j.1469-7998.1978.tb03316.x.

[ece372880-bib-0022] Heideman, P. D. , and R. C. B. Utzurrum . 2003. “Seasonality and Synchrony of Reproduction in Three Species of Nectarivorous Philippines Bats.” BMC Ecology 3, no. 1: 1–14. 10.1186/1472-6785-3-11. 14633285 PMC305358

[ece372880-bib-0023] Hodge, S. J. , M. B. V. Bell , and M. A. Cant . 2011. “Reproductive Competition and the Evolution of Extreme Birth Synchrony in a Cooperative Mammal.” Biology Letters 7, no. 1: 54–56. 10.1098/rsbl.2010.0555. 20685697 PMC3030886

[ece372880-bib-0024] Holand, O. , R. B. Weladji , H. Gjøstein , et al. 2004. “Reproductive Effort in Relation to Maternal Social Rank in Reindeer (*Rangifer tarandus*).” Behavioral Ecology and Sociobiology 57, no. 1: 69–76. 10.1007/s00265-004-0827-0.

[ece372880-bib-0025] Ims, R. A. 1990. “The Ecology and Evolution of Reproductive Synchrony.” Trends in Ecology & Evolution 5, no. 5: 135–140. 10.1016/0169-5347(90)90218-3. 21232341

[ece372880-bib-0026] Ims, R. A. , S. Bondrup‐Nielsen , and N. C. Stenseth . 1988. “Temporal Patterns of Breeding Events in Small Rodent Populations.” Oikos 53, no. 2: 229–234. 10.2307/3566067.

[ece372880-bib-0027] Lande, R. , S. Engen , and B. E. Sæther . 2003. Stochastic Population Dynamics in Ecology and Conservation. Oxford University Press.

[ece372880-bib-0028] Langefors, A. , D. Hasselquist , and T. von Schantz . 1998. “Extra‐Pair Fertilizations in the Sedge Warbler.” Journal of Avian Biology 29, no. 2: 134–144. 10.2307/3677191.

[ece372880-bib-0029] Loe, L. E. , C. Bonenfant , A. Mysterud , et al. 2005. “Climate Predictability and Breeding Phenology in Red Deer: Timing and Synchrony of Rutting and Calving in Norway and France.” Journal of Animal Ecology 74, no. 4: 579–588. 10.1111/j.1365-2656.2005.00987.x.

[ece372880-bib-0030] Marchand, P. , M. Garel , N. Morellet , et al. 2021. “A Standardised Biologging Approach to Infer Parturition: An Application in Large Herbivores Across the Hider‐Follower Continuum.” Methods in Ecology and Evolution 12, no. 6: 1017–1030. 10.1111/2041-210X.13584.

[ece372880-bib-0031] Marsden, A. D. , and K. L. Evans . 2004. “Synchrony, Asynchrony, and Temporally Random Mating: A New Method for Analyzing Breeding Synchrony.” Behavioral Ecology 15, no. 4: 699–700. 10.1093/beheco/arh057.

[ece372880-bib-0032] Matchinske, M. , S. Abades , L. A. Ebensperger , L. A. Correa , and L. D. Hayes . 2024. “Food Abundance and Group Size Influence the Phenology of Reproduction in Communally Breeding *Octodon degus* .” Behavioral Ecology and Sociobiology 78, no. 8: 87. 10.1007/s00265-024-03504-0.

[ece372880-bib-0033] Mattisson, J. , J. D. C. Linnell , O. Anders , et al. 2022. “Timing and Synchrony of Birth in Eurasian Lynx Across Europe.” Ecology and Evolution 12, no. 8: e9147. 10.1002/ece3.9147. 35923936 PMC9339757

[ece372880-bib-0034] Michel, E. S. , B. K. Strickland , S. Demarais , et al. 2020. “Relative Reproductive Phenology and Synchrony Affect Neonate Survival in a Nonprecocial Ungulate.” Functional Ecology 34, no. 12: 2536–2547. 10.1111/1365-2435.13680.

[ece372880-bib-0035] Ndibalema, V. G. 2009. “A Comparison of Sex Ratio, Birth Periods and Calf Survival Among Serengeti Wildebeest Sub‐Populations, Tanzania.” African Journal of Ecology 47, no. 4: 574–582. 10.1111/j.1365-2028.2008.00994.x.

[ece372880-bib-0036] Nussey, D. H. , T. H. Clutton‐Brock , D. A. Elston , S. D. Albon , and L. E. B. Kruuk . 2005. “Phenotypic Plasticity in a Maternal Trait in Red Deer.” Journal of Animal Ecology 74, no. 2: 387–396. 10.1111/j.1365-2656.2005.00941.x.

[ece372880-bib-0037] Nussey, D. H. , A. J. Wilson , and J. E. Brommer . 2007. “The Evolutionary Ecology of Individual Phenotypic Plasticity in Wild Populations.” Journal of Evolutionary Biology 20, no. 3: 831–844. 10.1111/j.1420-9101.2007.01300.x. 17465894

[ece372880-bib-0038] Ogutu, J. O. , N. Owen‐Smith , H. P. Piepho , and H. T. Dublin . 2015. “How Rainfall Variation Influences Reproductive Patterns of African Savanna Ungulates in an Equatorial Region Where Photoperiod Variation Is Absent.” PLoS One 10, no. 8: e0133744. 10.1371/journal.pone.0133744. 26295154 PMC4546645

[ece372880-bib-0039] Ogutu, J. O. , H. P. Piepho , H. T. Dublin , N. Bhola , and R. S. Reid . 2010. “Rainfall Extremes Explain Interannual Shifts in Timing and Synchrony of Calving in Topi and Warthog.” Population Ecology 52, no. 1: 89–102. 10.1007/s10144-009-0163-3.

[ece372880-bib-0040] Ordiz, A. , O. G. Støen , J. E. Swenson , I. Kojola , and R. Bischof . 2008. “Distance‐Dependent Effect of the Nearest Neighbor: Spatiotemporal Patterns in Brown Bear Reproduction.” Ecology 89, no. 12: 3327–3335. 10.1890/07-1921.1. 19137940

[ece372880-bib-0041] Paré, P. , C. Barrette , and J. Prescott . 1996. “Seasonal Reproduction of Captive Himalayan Tahrs (*Hemitragus jemlahicus*) in Relation to Latitude.” Journal of Mammalogy 77, no. 3: 826–832. 10.2307/1382688.

[ece372880-bib-0042] Philson, C. S. , C. Bruebach , T. Bastian , B. Barr , and D. T. Blumstein . 2024. “Timing of Seasonal Events Is Correlated With Social Network Position in a Wild Mammal.” Behavioral Ecology and Sociobiology 78, no. 5: 58. 10.1007/s00265-024-03472-5.

[ece372880-bib-0043] Plard, F. , J. M. Gaillard , T. Coulson , et al. 2014. “Long‐Lived and Heavier Females Give Birth Earlier in Roe Deer.” Ecography 37, no. 3: 241–249. 10.1111/j.1600-0587.2013.00414.x.

[ece372880-bib-0044] Plard, F. , N. G. Yoccoz , C. Bonenfant , F. Klein , C. Warnant , and J. M. Gaillard . 2015. “Disentangling Direct and Growth‐Mediated Influences on Early Survival: A Mechanistic Approach.” Journal of Animal Ecology 84, no. 5: 1363–1372. 10.1111/1365-2656.12378. 25882771

[ece372880-bib-0045] Robbins, C. T. , M. Ben‐David , J. K. Fortin , and O. L. Nelson . 2012. “Maternal Condition Determines Birth Date and Growth of Newborn Bear Cubs.” Journal of Mammalogy 93, no. 2: 540–546. 10.1644/11-MAMM-A-155.1.

[ece372880-bib-0046] Rotella, J. J. , J. T. Paterson , and R. A. Garrott . 2016. “Birth Dates Vary With Fixed and Dynamic Maternal Features, Offspring Sex, and Extreme Climatic Events in a High‐Latitude Marine Mammal.” Ecology and Evolution 6, no. 7: 1930–1941. 10.1002/ece3.1985. 27099704 PMC4831429

[ece372880-bib-0047] Russell, A. F. , and V. Lummaa . 2009. “Maternal Effects in Cooperative Breeders: From Hymenopterans to Humans.” Philosophical Transactions of the Royal Society, B: Biological Sciences 364, no. 1520: 1143–1167. 10.1098/rstb.2008.0298. PMC2666687 19324618

[ece372880-bib-0048] Rutberg, A. T. 1987. “Adaptive Hypotheses of Birth Synchrony in Ruminants: An Interspecific Test.” American Naturalist 130, no. 5: 692–710. 10.1086/284739.

[ece372880-bib-0049] Schmidt, J. , A. Kosztolányi , J. Tökölyi , et al. 2015. “Reproductive Asynchrony and Infanticide in House Mice Breeding Communally.” Animal Behaviour 101: 201–211. 10.1016/j.anbehav.2014.12.015.

[ece372880-bib-0050] Scott, I. C. , G. W. Asher , J. A. Archer , and R. P. Littlejohn . 2008. “The Effect of Conception Date on Gestation Length of Red Deer ( *Cervus elaphus* ).” Animal Reproduction Science 109, no. 1–4: 206–217. 10.1016/j.anireprosci.2007.11.025. 18178346

[ece372880-bib-0051] Sinclair, A. R. E. , S. A. R. Mduma , and P. Arcese . 2000. “What Determines Phenology and Synchrony of Ungulate Breeding in Serengeti?” Ecology 81, no. 8: 2100–2111. 10.2307/177099.

[ece372880-bib-0052] Stopher, K. V. , J. M. Pemberton , T. H. Clutton‐Brock , and T. Coulson . 2008. “Individual Differences, Density Dependence and Offspring Birth Traits in a Population of Red Deer.” Proceedings of the Royal Society B: Biological Sciences 275, no. 1647: 2137–2145. 10.1098/rspb.2008.0502. PMC2603214 18522909

[ece372880-bib-0053] Stutchbury, B. J. M. , E. S. Morton , and W. H. Piper . 1998. “Extra‐Pair Mating System of a Synchronously Breeding Tropical Songbird.” Journal of Avian Biology 29, no. 1: 72–78. 10.2307/3677343.

[ece372880-bib-0054] Thel, L. , S. Chamaillé‐Jammes , and C. Bonenfant . 2022. “How to Describe and Measure Phenology? An Investigation on the Diversity of Metrics Using Phenology of Births in Large Herbivores.” Oikos 2022, no. 4: e08917. 10.1111/oik.08917.

[ece372880-bib-0055] Turnley, M. T. , T. A. Hughes , R. T. Larsen , et al. 2024. “A Fine‐Scale Examination of Parturition Timing in Temperate Ungulates.” Ecology and Evolution 14, no. 7: e11703. 10.1002/ece3.11703. 38962024 PMC11222017

[ece372880-bib-0056] Varpe, O. 2017. “Life History Adaptations to Seasonality.” Integrative and Comparative Biology 57, no. 5: 943–960. 10.1093/icb/icx123. 29045732

[ece372880-bib-0057] Vindenes, Y. , S. Engen , and B. E. Sæther . 2008. “Individual Heterogeneity in Vital Parameters and Demographic Stochasticity.” American Naturalist 171, no. 4: 455–467. 10.1086/528965. 20374136

[ece372880-bib-0058] Zerbe, P. , M. Clauss , D. Codron , et al. 2012. “Reproductive Seasonality in Captive Wild Ruminants: Implications for Biogeographical Adaptation, Photoperiodic Control, and Life History.” Biological Reviews 87, no. 4: 965–990. 10.1111/j.1469-185X.2012.00238.x. 22780447

