# Supplementary material for: Parturition Synchrony Index: A Method for Assessing Individual Parturition Synchrony Within a Group or Population
Source: Ecol Evol. 2026 Jan 2;16(1):e72880. doi: 10.1002/ece3.72880 (PMC12758954; doi:10.1002/ece3.72880)
Supplement: Supplementary file 2 — Appendix S2: Figure S2.1 showing variation in PSI values across simulated groups covering all combinations of examined parameters. [file ECE3-16-e72880-s004.pdf]

APPENDIX S2

FIGURE S2.1 Variation in the PSI across the 40 simulated groups, each representing a unique combination of parturition season duration (10, 50, 100, 200, and 365 days) and group size (5, 10, 25, 50, 100, 500, 1,000, and 5,000 mothers) under 4 parturition date distributions: (a) lognormal, (b) normal, (c) bimodal, and (d) uniform. In each box plot, the open diamond indicates the mean, the horizontal line marks the median, the box spans the interquartile range (25th–75th percentiles), and the whiskers extend to the minimum and maximum non-outlier values. Open circles represent statistical outliers.

(a)

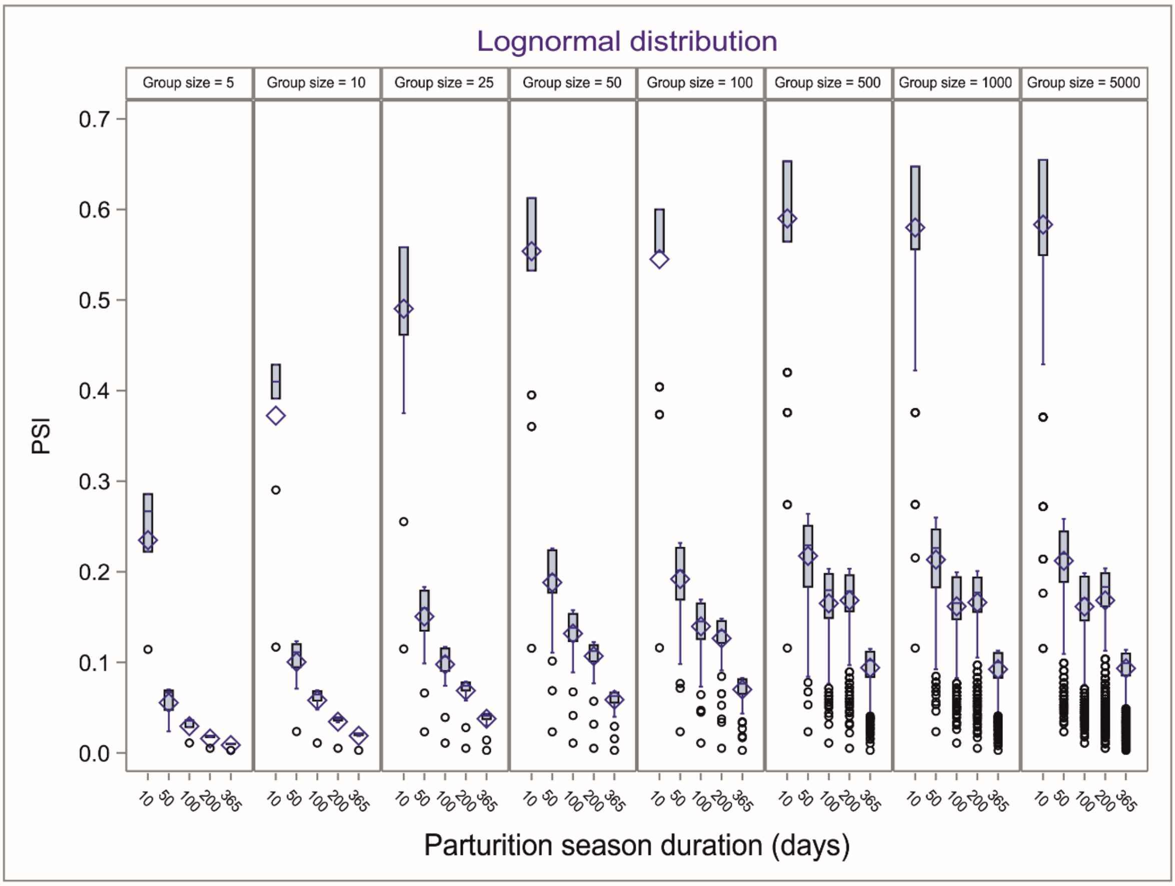
Lognormal distribution
Group size = 5
Group size = 10
Group size = 25
Group size = 50
Group size = 100
Group size = 500
Group size = 1000
Group size = 5000
PSI
0.7
0.6
0.5
0.4
0.3
0.2
0.1
0.0
10
50
100
200
365
Parturition season duration (days)

(b)

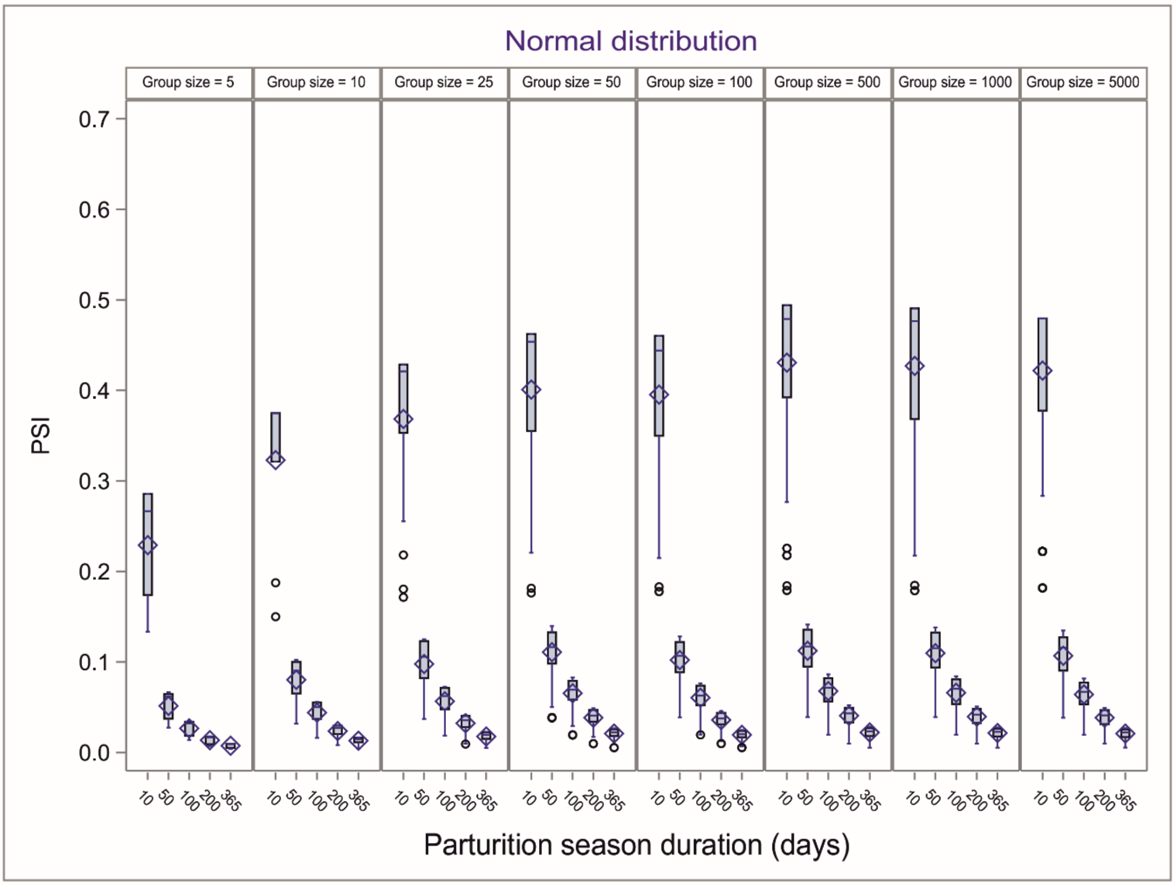
Normal distribution
Group size = 5
Group size = 10
Group size = 25
Group size = 50
Group size = 100
Group size = 500
Group size = 1000
Group size = 5000
PSI
0.7
0.6
0.5
0.4
0.3
0.2
0.1
0.0
10 50 100 200 365
Parturition season duration (days)

(c)

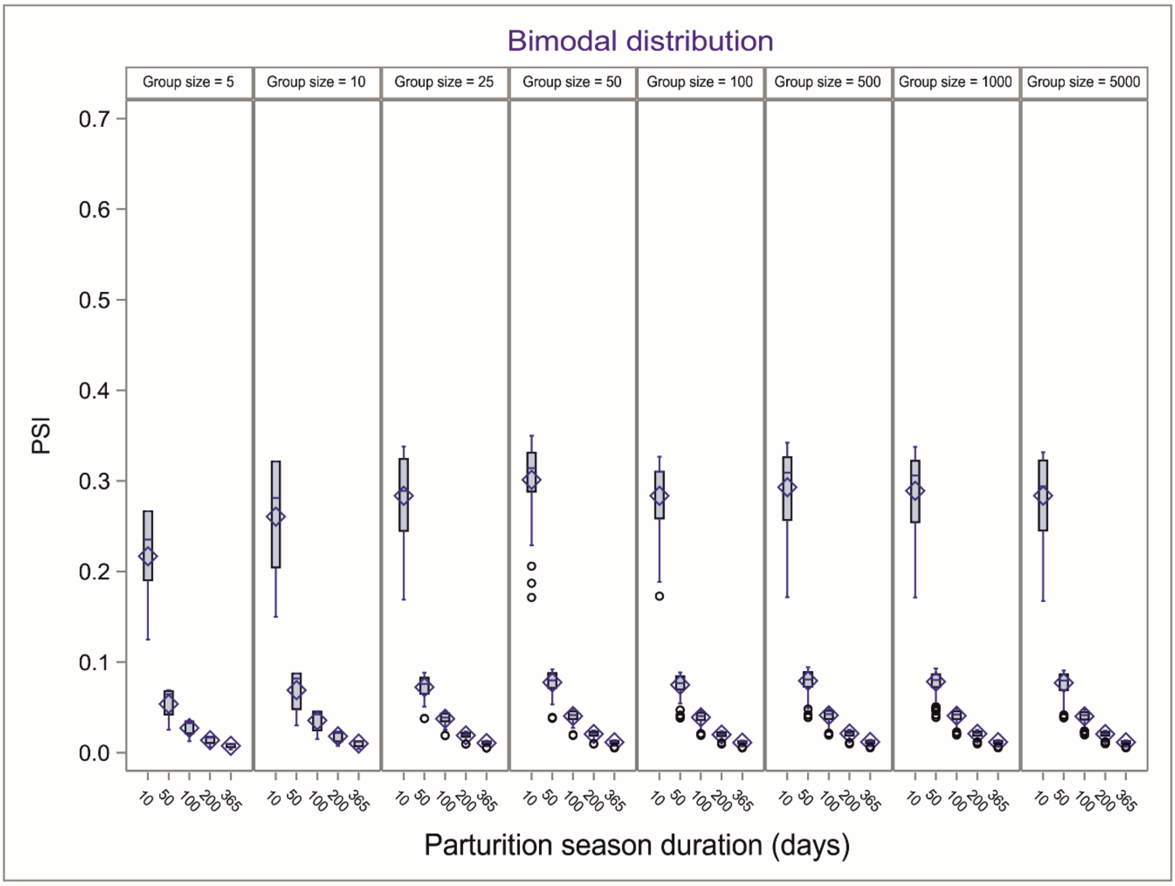
Bimodal distribution
Group size = 5
Group size = 10
Group size = 25
Group size = 50
Group size = 100
Group size = 500
Group size = 1000
Group size = 5000
PSI
0.7
0.6
0.5
0.4
0.3
0.2
0.1
0.0
10 50 100 200 365
Parturition season duration (days)

(d)

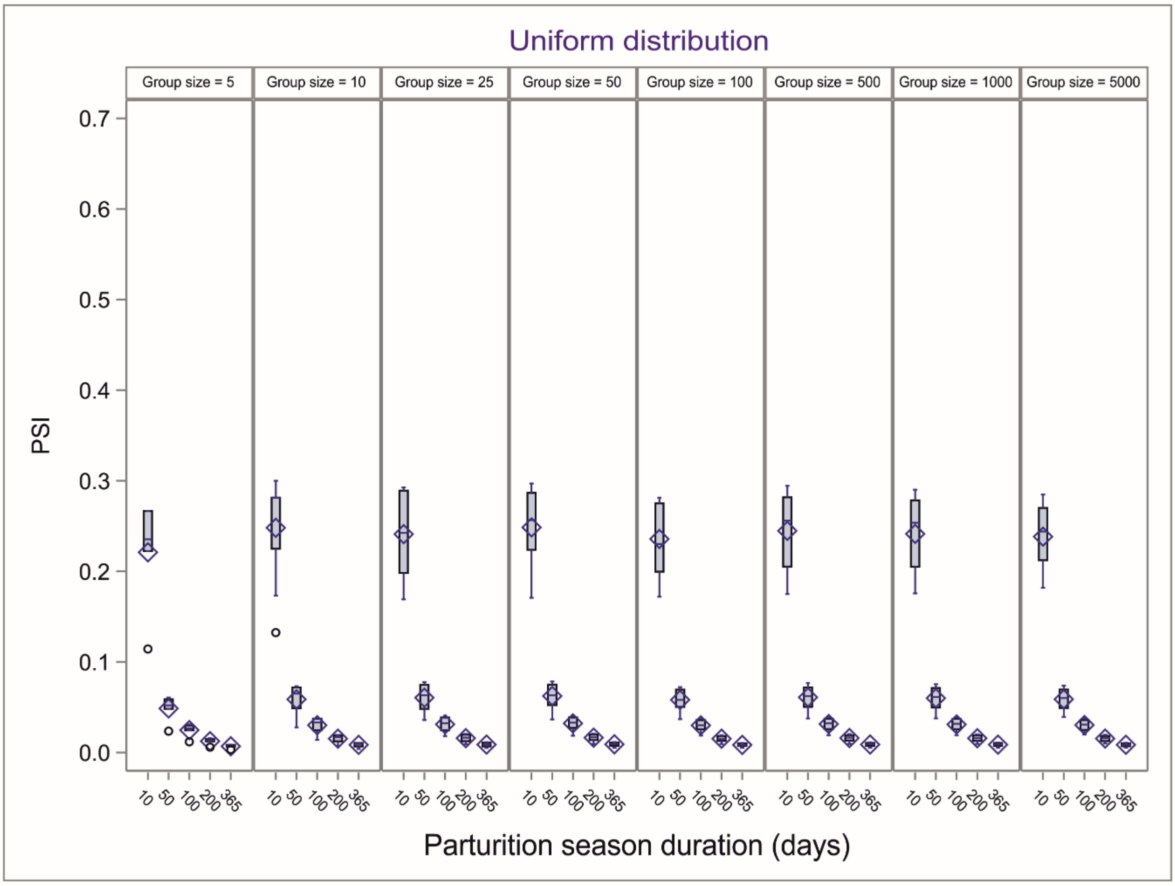

Uniform distribution
Group size = 5
Group size = 10
Group size = 25
Group size = 50
Group size = 100
Group size = 500
Group size = 1000
Group size = 5000
PSI
0.7
0.6
0.5
0.4
0.3
0.2
0.1
0.0
10
50
100
200
365
Parturition season duration (days)
