# Supplementary material for: Parturition Synchrony Index: A Method for Assessing Individual Parturition Synchrony Within a Group or Population
Source: Ecol Evol. 2026 Jan 2;16(1):e72880. doi: 10.1002/ece3.72880 (PMC12758954; doi:10.1002/ece3.72880)
Supplement: Supplementary file 3 — Appendix S3: Table S3.1 showing definitions of variables used and SAS and R code for calculating individual PSI values using an example data set. [file ECE3-16-e72880-s001.pdf]

APPENDIX S3

Contents

1. Table S3.1: Definitions of variables used in the calculation of individual PSI values
2. SAS code for calculating individual PSI values using an example dataset
3. R code for calculating individual PSI values using an example dataset

1. Table S3.1 Definitions of variables used in the calculation of individual PSI values.

Variable	Definition
Parturition_date	The parturition date of the focal mother in the group
Parturition_date2	The parturition date of the other mother in the group
PSI	Parturition synchrony index for an individual mother in the group
Days_diff	The absolute value of the difference between the parturition dates of the examined pair of mothers in the group
ID_mother	ID of the focal mother in the examined pair of mothers (ID_mother, ID_mother2) in the group
ID_mother2	ID of the other mother in the examined pair of mothers (ID_mother, ID_mother2) in the group
Ind, Ind2	The examined pair of mothers within the group (cross-tabulated pairwise); operational variables to facilitate working with ID_mother
No_mothers	The number of parturient mothers in the group
Relative_parturition_date	The difference between the parturition date of the focal mother (as indicated in the ID_mother column) and that of the first-birthing mother in the group (= Parturition_date - First_parturition_date + 1)
Year	The year of parturition for all mothers in the group

2. SAS code for calculating individual PSI values using an example dataset

```
/*Load dataset*/

DATA Dataset;
input Year ID_mother Parturition_date :ddmmyy10.;
format Parturition_date ddmmyy10.;
cards;
2009 200127 28-05-2009
2009 200206 01-06-2009
2009 200408 04-06-2009
2009 200124 08-06-2009
2009 200511 08-06-2009
2009 199712 10-06-2009
2009 200402 10-06-2009
2009 200111 12-06-2009
2009 200010 15-06-2009
2009 200121 15-06-2009
2009 200416 15-06-2009
2009 200523 17-06-2009
2009 200514 18-06-2009
2009 199718 23-06-2009
2009 199725 23-06-2009
2009 200113 23-06-2009
2009 200411 23-06-2009
2009 200204 25-06-2009
2009 200005 29-06-2009
2010 200010 31-05-2010
2010 200206 31-05-2010
2010 200503 31-05-2010
2010 199712 03-06-2010
2010 200005 03-06-2010
2010 200127 03-06-2010
2010 200511 03-06-2010
2010 200514 03-06-2010
2010 200111 08-06-2010
2010 200402 08-06-2010
2010 199718 11-06-2010
2010 200113 11-06-2010
2010 200408 11-06-2010
2010 200411 11-06-2010
2010 200416 11-06-2010
2010 200121 14-06-2010
2010 200124 14-06-2010
2010 200523 14-06-2010
2010 200204 21-06-2010
2016 200411 30-05-2016
2016 200915 30-05-2016
2016 200402 01-06-2016
2016 200909 01-06-2016
2016 201207 01-06-2016
2016 200127 03-06-2016
2016 200523 06-06-2016
2016 201411 06-06-2016
2016 201417 06-06-2016
2016 201205 06-06-2016
2016 200408 09-06-2016
2016 201212 09-06-2016
2016 200206 10-06-2016
2016 200111 14-06-2016
2016 200514 14-06-2016
2016 200503 14-06-2016
```

```
2016 200416 14-06-2016
2016 200904 16-06-2016
2016 200113 20-06-2016
2018 200915 01-05-2018
2018 201605 07-05-2018
2018 200909 28-05-2018
2018 201520 28-05-2018
2018 201610 28-05-2018
2018 201612 30-05-2018
2018 201411 01-06-2018
2018 201412 01-06-2018
2018 200402 05-06-2018
2018 200514 05-06-2018
2018 200408 05-06-2018
2018 201417 07-06-2018
2018 201606 07-06-2018
2018 200411 11-06-2018
2018 200904 11-06-2018
2018 201212 11-06-2018
2018 201601 11-06-2018
2018 201507 13-06-2018
2018 201618 27-06-2018
;
run;


/*No_mothers and Relative_parturition_date generated*/

proc sql;
create table A as
select *, (Parturition_date - min(Parturition_date)) + 1 as
Relative_parturition_date, count(ID_mother) as No_mothers
from Dataset
group by Year order by Year, Parturition_date;
quit;

/*Preliminary cross table with ID_mother numbered in group - Ind column*/

DATA A1;
set A;
by Year;
retain Ind .;
Ind + 1;
if first.year then Ind = 1;
run;

/*Preliminary cross table for generating pairs of mothers in group - Ind,
Ind2 columns*/

DATA A2;
set A1;
do Ind2 = 1 to No_mothers; output; end;
run;

/*Preliminary table for merging pairs of mothers - information about other
mother of the pair*/

DATA help_Ind2;
set A1;
ID_mother2 = ID_mother;
format Parturition_date2 ddmmyy10.;
Parturition_date2 = Parturition_date;
```

```
Ind2 = Ind;
keep Year ID_mother2 Parturition_date2 Ind2;
run;

/*Merging information about both mothers of the pair*/

%let BY = Year Ind2;
proc sort data = A2; by &by; proc sort data = help_Ind2; by &by;
DATA A3;
merge A2 help_Ind2;
by &by;
Days_diff = abs(Parturition_date2 - Parturition_date);
if Ind ne Ind2;
run;
proc sort data = A3; by Year Ind Ind2;
run;

/*Total_no_days - PSI formula numerator*/

proc sql;
create table A4 as
select *, 1 / (sum(Days_diff + 1) / (No_mothers - 1)) as PSI
from A3
group by Year, Ind order by Year, Parturition_date, ID_mother, ID_mother2;
quit;

/*Final table with individual PSIs*/

proc sql;
create table PSI as
select distinct *
from A4 (drop = Ind -- Days_diff)
group by Year, ID_mother order by Year, Parturition_date, ID_mother;
quit;

/*Print final table with individual PSIs*/

title "Final table with individual PSIs";
proc print;
run;
```

3. R code for calculating individual PSI values using an example dataset

Note. Ensure that the lubridate, dplyr, and tidyr packages are installed before executing the R code.

```
# Load necessary libraries

library(lubridate)
library(dplyr)
library(tidyr)

# Load dataset

Dataset <- read.table(header = TRUE, text = "
Year ID_mother Parturition_date0
2009 200127 '28-05-2009'
2009 200206 '01-06-2009'
2009 200408 '04-06-2009'
2009 200124 '08-06-2009'
2009 200511 '08-06-2009'
2009 199712 '10-06-2009'
2009 200402 '10-06-2009'
2009 200111 '12-06-2009'
2009 200010 '15-06-2009'
2009 200121 '15-06-2009'
2009 200416 '15-06-2009'
2009 200523 '17-06-2009'
2009 200514 '18-06-2009'
2009 199718 '23-06-2009'
2009 199725 '23-06-2009'
2009 200113 '23-06-2009'
2009 200411 '23-06-2009'
2009 200204 '25-06-2009'
2009 200005 '29-06-2009'
2010 200010 '31-05-2010'
2010 200206 '31-05-2010'
2010 200503 '31-05-2010'
2010 199712 '03-06-2010'
2010 200005 '03-06-2010'
2010 200127 '03-06-2010'
2010 200511 '03-06-2010'
2010 200514 '03-06-2010'
2010 200111 '08-06-2010'
2010 200402 '08-06-2010'
2010 199718 '11-06-2010'
2010 200113 '11-06-2010'
2010 200408 '11-06-2010'
2010 200411 '11-06-2010'
2010 200416 '11-06-2010'
2010 200121 '14-06-2010'
2010 200124 '14-06-2010'
2010 200523 '14-06-2010'
2010 200204 '21-06-2010'
2016 200411 '30-05-2016'
2016 200915 '30-05-2016'
2016 200402 '01-06-2016'
2016 200909 '01-06-2016'
2016 201207 '01-06-2016'
2016 200127 '03-06-2016'
2016 200523 '06-06-2016'
```

```
2016 201411 '06-06-2016'
2016 201417 '06-06-2016'
2016 201205 '06-06-2016'
2016 200408 '09-06-2016'
2016 201212 '09-06-2016'
2016 200206 '10-06-2016'
2016 200111 '14-06-2016'
2016 200514 '14-06-2016'
2016 200503 '14-06-2016'
2016 200416 '14-06-2016'
2016 200904 '16-06-2016'
2016 200113 '20-06-2016'
2018 200915 '01-05-2018'
2018 201605 '07-05-2018'
2018 200909 '28-05-2018'
2018 201520 '28-05-2018'
2018 201610 '28-05-2018'
2018 201612 '30-05-2018'
2018 201411 '01-06-2018'
2018 201412 '01-06-2018'
2018 200402 '05-06-2018'
2018 200514 '05-06-2018'
2018 200408 '05-06-2018'
2018 201417 '07-06-2018'
2018 201606 '07-06-2018'
2018 200411 '11-06-2018'
2018 200904 '11-06-2018'
2018 201212 '11-06-2018'
2018 201601 '11-06-2018'
2018 201507 '13-06-2018'
2018 201618 '27-06-2018'
")

# Convert Parturition_date to Date format

Dataset$Parturition_date <- dmy(Dataset$Parturition_date0)

# No_mothers and Relative_parturition_date generated

A <- Dataset %>%
group_by(Year) %>%
mutate(Relative_parturition_date = as.numeric(Parturition_date -
min(Parturition_date)) + 1,
No_mothers = n()) %>%
arrange(Year, Parturition_date)

# Preliminary cross table with ID_mother numbered in group - Ind column

A1 <- A %>%
group_by(Year) %>%
mutate(Ind = row_number()) %>%
ungroup()

# Preliminary cross table for generating pairs of mothers in group - Ind,
Ind2 columns

A2 <- A1 %>%
rowwise() %>%
mutate(Ind2 = list(1:No_mothers)) %>%
unnest(Ind2)
```

```
# Preliminary table for merging pairs of mothers - information about other
mother of the pair

help_Ind2 <- A1 %>%
select(Year, ID_mother2 = ID_mother, Parturition_date2 = Parturition_date,
Ind2 = Ind)


# Merging information about both mothers of the pair

A3 <- A2 %>%
left_join(help_Ind2, by = c("Year", "Ind2")) %>%
mutate(Days_diff = as.integer(abs(as.numeric(Parturition_date2 -
Parturition_date)))) %>%
filter(Ind != Ind2) %>%
arrange(Year, Ind, Ind2)


# Total_no_days - PSI formula numerator

A4 <- A3 %>%
group_by(Year, Ind) %>%
mutate(PSI = 1 / (sum(Days_diff + 1) / (No_mothers - 1))) %>%
ungroup() %>%
arrange(Year, Parturition_date, ID_mother, ID_mother2)


# Final table with individual PSIs

PSI <- A4 %>%
select(-Parturition_date0, -Ind, -Ind2, -ID_mother2, -Parturition_date2, -
Days_diff) %>%
group_by(Year, ID_mother) %>%
arrange(Year, Parturition_date, ID_mother) %>%
distinct() %>%
ungroup()

# Print final table with individual PSIs

print(PSI, n = Inf)
```
